# Supplementary material for: Empirical methods for controlling false positives and estimating confidence in ChIP-Seq peaks
Source: BMC Bioinformatics. 2008 Dec 5;9:523. doi: 10.1186/1471-2105-9-523 (PMC2628906; doi:10.1186/1471-2105-9-523)
Supplement: Additional File 1 — A variety of html documents from the USeq web site detailing the available applications, their best usage, output file type descriptions, command line menus, etc. [file 1471-2105-9-523-S1.zip › USeqUserGuides/JavaDoc.zip!/JavaDoc/bioroot/package-use.html]

Uses of Package bioroot


|  |  |  |
| --- | --- | --- |
| **Overview** \| **Package** \| Class \| **Use** \| **Tree** \| **Deprecated** \| **Index** \| **Help** | |  |
| PREV   NEXT | **FRAMES**    **NO FRAMES**     **All Classes** |  |


---


## **Uses of Package bioroot**

| Packages that use bioroot | |
| --- | --- |
| **bioroot** |  |
| **bioroot.antibody** |  |
| **bioroot.oligo** |  |
| **bioroot.plasmid** |  |
| **bioroot.strain** |  |

Classes in bioroot used by bioroot

****DBUtil****
  
          Utility class to hold database relevant methods and fields, some from Hall and Brown
The following is a collection of stuff for interacting with a SQL database
First open a connection by calling connectMySQL, then get results with
executeSQL, then close the connection with closeConnection.

****Feature****

****IdNameMatch****

****LabGroup****

****LoginBean****

****Organization****
  
          Container for holding info about Organization membership.

****PatternMatch****
  
          Class to hold information and helper methods relating to pattern matches.

****Preference****

****ReagentBean****

****SearchHit****
  
          Holds info relating to a search hit from a database LIKE query.

****UserBean****
  
          User information.

Classes in bioroot used by bioroot.antibody

****DBUtil****
  
          Utility class to hold database relevant methods and fields, some from Hall and Brown
The following is a collection of stuff for interacting with a SQL database
First open a connection by calling connectMySQL, then get results with
executeSQL, then close the connection with closeConnection.

****Feature****

****ReagentBean****

****UserBean****
  
          User information.

Classes in bioroot used by bioroot.oligo

****DBUtil****
  
          Utility class to hold database relevant methods and fields, some from Hall and Brown
The following is a collection of stuff for interacting with a SQL database
First open a connection by calling connectMySQL, then get results with
executeSQL, then close the connection with closeConnection.

****IdNameMatch****

****Preference****

****ReagentBean****

****UserBean****
  
          User information.

Classes in bioroot used by bioroot.plasmid

****BlastMatcher****

****DBUtil****
  
          Utility class to hold database relevant methods and fields, some from Hall and Brown
The following is a collection of stuff for interacting with a SQL database
First open a connection by calling connectMySQL, then get results with
executeSQL, then close the connection with closeConnection.

****IdNameMatch****

****ReagentBean****

****UserBean****
  
          User information.

Classes in bioroot used by bioroot.strain

****DBUtil****
  
          Utility class to hold database relevant methods and fields, some from Hall and Brown
The following is a collection of stuff for interacting with a SQL database
First open a connection by calling connectMySQL, then get results with
executeSQL, then close the connection with closeConnection.

****IdNameMatch****

****Preference****

****ReagentBean****

****UserBean****
  
          User information.

---


|  |  |  |
| --- | --- | --- |
| **Overview** \| **Package** \| Class \| **Use** \| **Tree** \| **Deprecated** \| **Index** \| **Help** | |  |
| PREV   NEXT | **FRAMES**    **NO FRAMES**     **All Classes** |  |


---
